# Efficacy of radiofrequency microdebridement (TOPAZ) in tendinopathy: a systematic review and meta-analysis of randomised clinical trials

**DOI:** 10.1186/s13018-026-06827-y

**Published:** 2026-03-31

**Authors:** Cheryl Loh, Stella Polzer, Neal Millar, Dimitris Challoumas

**Affiliations:** School of Infection and Immunity, College of Medical, Veterinary and Life Sciences, University of Glasgow, Sir Graeme Davies Building, 120 University Place, Glasgow, G12 8TA UK

**Keywords:** Coblation, Tendinitis, Achilles, Tennis elbow, Subacromial impingement, GTPS

## Abstract

**Objectives:**

The aim of our systematic review and meta-analysis was to assess the outcomes associated with the use of radiofrequency microdebridement (RM; TOPAZ) as compared to other interventions in all tendinopathies.

**Methods:**

We searched multiple databases for RCTs reporting efficacy outcomes associated with the use of RM in any type of tendinopathy, compared to any other treatment or no treatment. Outcome measures of interest included patient-reported pain visual analogue scale (VAS, primary outcome), patient-reported function and complications (secondary outcomes). Outcomes were pooled separately at short- (up to 12 weeks), mid- (13–52 weeks) and long-term (more than 52 weeks) follow-up. Risk of bias of each study and certainty of evidence of each result were assessed using the Cochrane Collaboration and GRADE tools, respectively.

**Results:**

Nine RCTs with a total of 318 patients were included. For the primary outcome, when comparing RM with surgical release, no statistically significant differences were found at any follow-up time point when all tendinopathies were combined [lateral elbow, shoulder, Achilles; short-term mean difference, MD, 0.32, 95% confidence interval (-1.19 to 1.83); mid-term MD 0.09 (-0.22 to 0.40); long-term MD -0.04 (-0.30 to 0.23)] or when lateral elbow tendinopathy, shoulder and Achilles tendinopathies were analysed separately (low certainty evidence). When comparing RM with physiotherapy in Achilles tendinopathy, RM was statistically superior for pain at long-term follow-up [MD -3.50 (-4.90 to -2.10), low certainty, single study]. Finally, when RM was added to surgical release in shoulder and gluteal tendinopathy, there were no significant pain-relieving benefits compared to surgical release alone at short-, mid- or long-term follow-up for shoulder, and long-term follow-up for gluteal. Some functional short- and mid-term benefits were observed with the use of RM in some tendinopathies.

**Conclusion:**

Based on the existing evidence, the use of RM is associated with generally equivalent outcomes compared to surgical release for the included tendinopathies. A formal cost analysis may be useful in the future for definitive conclusions, as well as more high-quality RCTs.

**Supplementary Information:**

The online version contains supplementary material available at 10.1186/s13018-026-06827-y.

## Introduction

Tendinopathy is an umbrella term encapsulating pathologies that most commonly derive due to overuse, though its exact intrinsic mechanisms remain unclear. The relationship between repetitive motion/tendon strain resulting in microvascular/nerve degeneration and subsequent remodelling of the extracellular matrix have been favourably theorised, as well as has the role of inflammation, the effects of failed tendon healing and abnormal neoinnervation [1–3]. In general populations, tendinopathy is expected to affect 1–2% of adults in their lifetime; most commonly: rotator cuff (5.5%) and elbow (1.3%) tendinopathy of the upper limb and Achilles (2.4%) and tibialis posterior (2.4%) tendinopathies of the lower limb [4].

The diagnosis of tendinopathy is predominantly clinical. Factors that influence choice of treatment include physician and patient preferences, chronicity of symptoms, level of activity, co-morbidities and previously trialled treatments. Non-invasive approaches include physiotherapy for tendon rehabilitation, extracorporeal shock wave therapy, NSAIDs and injections (corticosteroid, hyaluronic acid, platelet rich plasma etc.) [5]. Surgery is indicated only after extensive non-surgical management fails and it aims to remove fibrotic adhesions, restore vascularity, and disrupt neoinnervation.

Recently, radiofrequency microdebridement (RM) is gaining popularity for the management of tendinopathy resistant to non-invasive measures however the evidence on its efficacy compared to other treatment modalities remains limited. RM or ablation, also referred to as TOPAZ, utilises a wand to create microscopic traumatic holes in scarred tendons to reintroduce blood flow and it aims to reinitiate the healing process without damage to surrounding tissue. It is generally used after an unsuccessful trial of conservative methods and it could be performed either in the operating theatre or in the office, with aseptic precautions.

This systematic review and meta-analysis aims to present the findings of published randomised controlled trials (RCTs) assessing the use of TOPAZ as compared to all other interventions in all tendinopathies. Our research hypothesis was that RM is not superior to other interventions in tendinopathy.

## Methods

We conducted and authored the present systematic review according to the Preferred Reporting Items for Systematic Reviews and Meta-Analyses (PRISMA) guidelines [6]. A protocol was written and registered on the PROSPERO website (CRD42023471620).

### Eligibility

Studies were eligible if they had a randomised design and compared TOPAZ to any other treatment, including no treatment or placebo, in patients with any type of tendinopathy. No criteria were applied for publication year, severity of tendinopathy or previously trialled treatments. Non-English studies, case reports, reviews, studies in non-adult patients and studies in insertional Achilles tendinopathy (pathophysiologically different condition to mid-portion tendinopathy) were excluded.

### Search strategy

We conducted a thorough literature search via Medline, EMBASE and the Cochrane Database from inception to March 2025. We used the following search terms in “all fields”: “((TOPAZ) OR (radiofrequency)) OR (coblation) OR (ablation) OR (*debridement) OR (*tenotomy) AND ((tendinopathy) OR (tendinitis)) OR ((tendinosis) OR (Achilles)) OR (patellar) OR (jumper’s knee) OR (rotator cuff impingement) OR tennis elbow OR (greater trochanteric pain syndrome) OR (gluteal) OR (GTPS) OR (trochanteric bursitis)”.

We screened relevant review articles to identify eligible articles that may have been missed at the initial search. Additionally, reference list screening and citation tracking in Google Scholar were performed for each eligible article. The grey literature was searched via Open Grey for unpublished studies to minimise the risk of publication bias.

For the purposes of this review, the terms ‘microdebridement’, ‘ablation’, ‘coblation’, and ‘TOPAZ’ have been used interchangeably.

### Screening

After removing duplicates from the initial search, we performed title and then abstract screening, followed by full-text screening to confirm article eligibility, by two reviewers (CL, DC) independently. Discrepancies were resolved with involvement of a third author (NM).

### Data extraction–handling

We tabulated the key methodological characteristics and results of each included study in Microsoft Word (two reviewers independently, CL and DC) to facilitate analysis and presentation.

Our primary outcomes were patient-reported pain. Our secondary outcomes were patient-reported function and complications. Follow-up time periods were pre-defined as short-term (up to and including 12 weeks), mid-term (13 weeks to 52 weeks) and long-term (> 52 weeks).

We performed quantitative analyses, in the form of pairwise meta-analyses, where two or more RCTs compared the outcomes of the same two interventions and used the same outcome measures, provided that there was no substantial clinical heterogeneity, (populations and interventions across pooled studies had to be similar)

Risk of bias was assessed using the Cochrane Collaboration tool by two authors independently (DC and CL), and disagreements were resolved with involvement of a third author (NM) [7]. Certainty of evidence was assessed by the senior author with the use of the GRADE tool [8, 9]. For pain VAS, DASH, Constant score and VISA-A, clinical significance was set based on Challoumas et al. (2023) ; for WOMAC and FAOS, it was set at 10 points [10, 11].

### Statistical analysis

We used the Review Manager V.5 (RevMan) software for pairwise meta-analyses and their accompanying forest plots, p values and heterogeneity tests (Chi^2^ and I^2^). Mean differences (MD) with 95% confidence intervals were calculated for continuous and dichotomous outcomes, respectively. Where numerical results of different scales were pooled in meta-analyses for the same outcome, standardised mean differences (SMDs) instead of MDs were used (e.g. in function). SMDs can be converted back to a MD of each scale by multiplying it by the pooled standard deviation associated with the use of that scale. Alternatively, they can simply be interpreted by using the Cohen’s d rule of thumb (0.2–0.5 = small effect, 0.5–0.8 = medium effect, > 0.8 = large effect) [12]. Expecting wide variability in studies’ settings, random-effects models were deployed for meta-syntheses. Publication bias was not assessed as no comparison included more than 10 studies. Where heterogeneity was high (I^2^ > 75%), the responsible studies were identified and removed through sensitivity analyses and the meta-analyses were re-ran without them, where at least 3 studies were included in meta-analyses [9].

## Results

Nine [9] RCTs (10 publications) with a total of 318 patients with tendinopathy (mean age 38 years) were found to be eligible [13–22] assessing the use of RM in the following tendinopathies: lateral elbow (*n* = 3 RCTs, 4 publications), shoulder (*n* = 3 studies), Achilles (*n* = 2 studies) and greater trochanteric pain syndrome (GTPS, *n* = 1 study). The additional publication reported long-term results of the same study. The article selection process can be seen in the PRISMA flow chart (Suppl. Figure 1) and the study characteristics are summarised in Table 1, along with the results of their risk of bias assessment in Suppl. Table 1. No additional articles were identified from reviews, reference list screening, citation tracking or the grey literature.

**Table 1:** Population, intervention and outcome measure characteristics of the included RCTs

First Author Pubmed ID	Pathology	Population (*n*, mean age)	Intervention	Duration of symptoms	Follow Up	Outcome Measure
Morrison et al. [20] 28,495,412	Achilles tendinopathy	*N* = 36 (47.5 years)	RM *n* = 20 vs. Open surgical release *n* = 16	Unknown	0, 6 months	VISA-A Pain VAS
Al-Ani et al. [19] 36,345,020	Achilles tendinopathy	*N* = 38 (45.5 years)	RM *n* = 20 vs. Physiotherapy *n* = 18	RM group: 2.5+/-2.3yrs Physiotherapy group: 2.4+/-1.3yrs	0, 1year, 2years	Pain VAS FAOS MRI
Blakey et al. [21] 32,047,828	Greater trochanteric pain syndrome	*N* = 33 (57.7 years)	Arthroscopic gluteal bursectomy/Iliotibial band release *n* = 17 vs. Arthroscopic gluteal bursectomy/Iliotibial band release + RM *n* = 16	All minimum 6 months	0, 6 weeks, 12 weeks, 24 weeks, 52 weeks	MHHS Pain VAS WOMAC SF12
Meknas et al. [16] 18,559,469	Lateral elbow tendinopathy	*N* = 24 (48 years)	Open extensor tendon surgical release *n* = 11 vs. RM *n* = 13	Surgical group: 27.6 months RM: 22 months All minimum 12 months	0, 3 weeks, 6 weeks, 12 weeks, 10–18 months	Pain VAS Grip strength MEPS
Lee et al. [14] 29,366,739	Lateral elbow tendinopathy	*N* = 46 (51.4 years)	Arthroscopic extensor tendon surgical release *n* = 24 vs. RM *n* = 22	Surgical group: 26.17+/-8.14 months RM group: 23.91+/-6.98 months	0, 3 months, 6 months, 12 months, 24 months	Pain VAS DASH MEPS Grip power Flexion extension arc
Hamlin et al. [15] 29,276,537	Lateral elbow tendinopathy	*N* = 41 (47.8 years)	Open extensor tendon surgical release *n* = 18 vs. RM *n* = 23	Unknown	0, 6 weeks, 6 months, 12 months	Pain NRS DASH Grip strength
Meknas et al. [13] 26,535,247	Lateral elbow tendinopathy	*N* = 24 (48 years)	Open extensor tendon surgical release *n* = 11 vs. RM *n* = 13	Surgical group: 28 month s RM group: 22 months All minimum 1yr	0, 5–7 years	MEPS MRI Pain VAS Strength
Lu et al. [17] 23,994,459	Shoulder tendinopathy	*N* = 80 (49.7 years)	ASD *n* = 40 vs. ASD + RM *n* = 40	15.2 months	0, 3 weeks, 6 weeks, 3 months, 6 months, 1 year	Pain VAS ASES UCLA Constant-Murley SST ROM
Taverna et al. [18] 17,916,468	Shoulder tendinopathy	*N* = 60 (52.6 years)	ASD *n* = 30 vs. RM n-30	Unknown	0, 6 weeks, 3 months, 6 months, 12 months	ASES Pain VAS Constant-Murley UCLA
Al-Ani et al. [22] 31,473,769	Shoulder tendinopathy	*N* = 27 (49.0 years)	ASD *n* = 14 vs. RM *n* = 13	Surgical group: 12.4months RM group: 13.1 months	0, 12 weeks, 6 months, 2 years	Pain VAS Constant-Murley Strength MRI (tendinosis grade, thickness of supraspinatus tendon)

ASES, American Shoulder and Elbow Surgeon score; DASH, disabilities of the arm, shoulder and hand scale; FAOS, foot and ankle outcomes score; MEPS, mayo elbow performance index; MHHS, modified Harris hip score; MRI, magnetic resonance imaging; NRS, numerical rating scale; SF-12, short-form 12; SST, simple shoulder test; UCLA, university of California Los Angeles score; VAS, visual analogue scale; VISA-A, Victorian Institute of Sport Assessment - Achilles

Overall risk of bias was ‘high’ in all 9 studies, mostly due to blinding concerns. Publication years ranged from 2007 to 2021. Overall, RM and its efficacy in managing different tendinopathies was studied against physiotherapy and surgical approaches (including both open and arthroscopic tendon release as well as a single paper exploring its efficacy against gluteal bursectomy).

In the results presented below, negative MD values favour RM and positive values favour the comparator intervention.

### Surgical release vs. TOPAZ

#### All tendinopathies

**Fig. 1:**
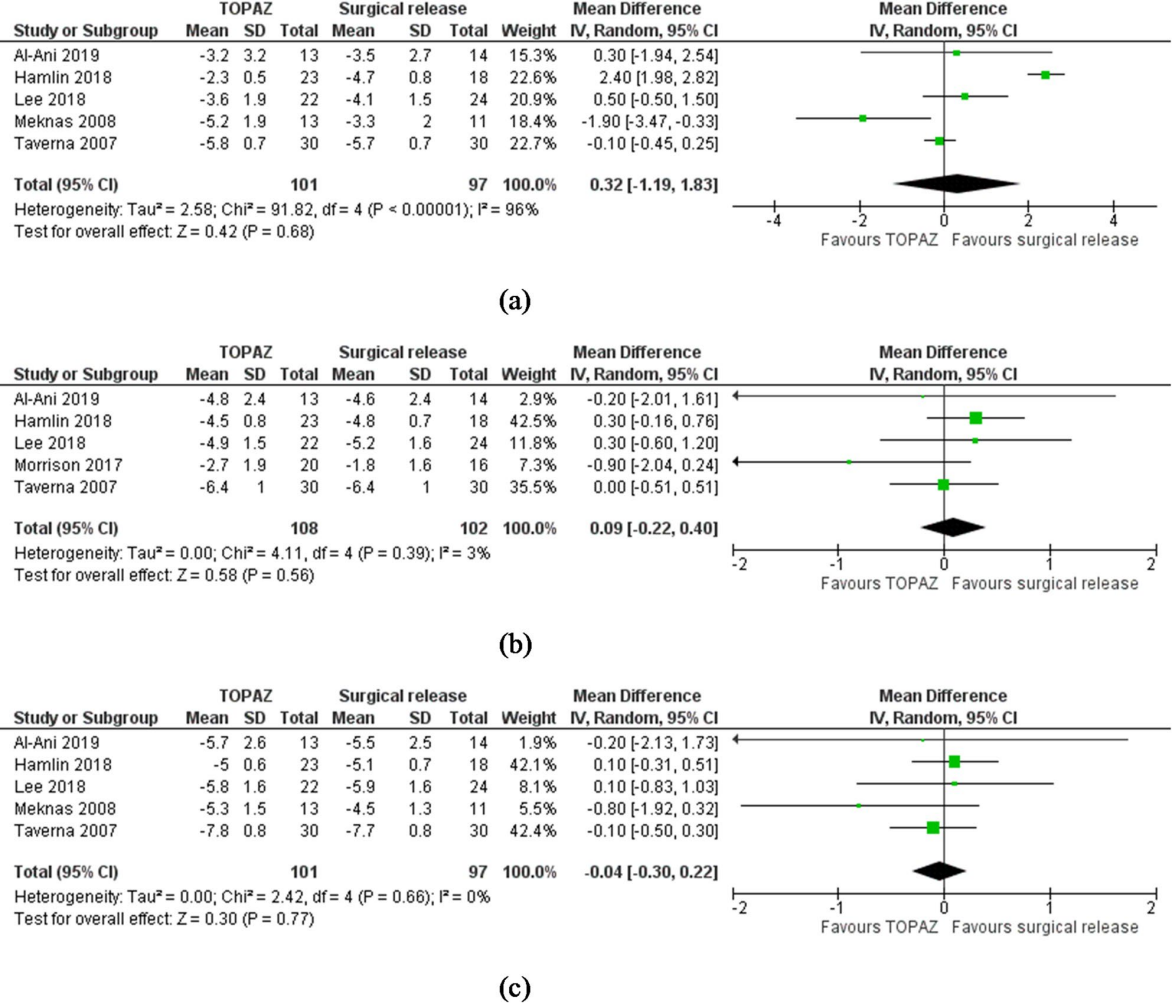
**a** Meta-analysis results showing forest plot and accompanying heterogeneity test for the comparison between radiofrequency microdebridement and surgical release (open or arthroscopic) for all tendinopathies combined for short-term pain visual analogue scale (0–10). **b** Meta-analysis results showing forest plot and accompanying heterogeneity test for the comparison between radiofrequency microdebridement and surgical release (open or arthroscopic) for all tendinopathies combined for mid-term pain visual analogue scale (0–10). **c** Meta-analysis results showing forest plot and accompanying heterogeneity test for the comparison between radiofrequency microdebridement and surgical release (open or arthroscopic) for all tendinopathies combined for long-term pain visual analogue scale (0–10)

Patient-reported pain (VAS): Six studies were eligible for these meta-analyses [13–16, 18, 22]. Three of them included patients with lateral elbow, two with shoulder and one with Achilles tendinopathy. Overall, compared to surgical release, patient reported pain for this group was associated with no statistically significant differences at any follow-up time period [short-term, MD 0.32, 95% CI (-1.19 to 1.83), I^2^ = 96%, *P* = 0.68, *n*=5RCTs, GRADE = low, Fig. 1a]; mid-term, MD 0.09 (-0.22 to 0.40), I^2^ = 3%, *P* = 0.56, *n* = 5, GRADE = low, Fig. 1b] and long-term, MD − 0.04(-0.30 to 0.23), I^2^ = 0%, *N* = 5, *P* = 0.77, GRADE = low, Fig. 1c]. For short-term pain, when the studies responsible for the high statistical heterogeneity were removed, the result was similar [MD 0.03 (-0.36 to 0.30), I^2^ = 0%, *n* = 3, *P* = 0.88, GRADE = low].

Function: Six RCTs (shoulder *n* = 2, lateral elbow *n* = 3, Achilles *n* = 1) and the following function scales participated in these meta-analyses: Constant-Murley (*n* = 2), DASH (*n* = 2), VISA-A (*n* = 1), MEPS (*n* = 1). At short-term and mid-term follow-up, results favoured RM at statistical significance compared to surgical release [SMD 0.37(0.06 to 0.68), I^2^ = 14%, *n* = 5, *P* = 0.02, GRADE = low, suppl. Figure 2a; SMD 0.64 (0.09 to 1.20), I^2^ = 73%, *n* = 5, *P* = 0.02, GRADE = low, suppl. Figure 2b, respectively]. For mid-term follow-up, when the analysis was re-ran without the study that was responsible for the high statistical heterogeneity, RM was still superior at statistical significance [SMD 0.38 (0.01 to 0.74), I^2^ = 27%, *n* = 4, *P* = 0.02, GRADE = low]. At long-term follow-up, no statistical significance was observed between the two interventions [SMD 0.56 (-0.26 to 1.38), I^2^ = 85%, *n* = 4, *P* = 0.18, GRADE = low, suppl. Figure 2c). This result did not change when the meta-analysis was re-ran without the study responsible for the high statistical heterogeneity [SMD 0.10 (-0.24 to 0.44), I^2^ = 0%, *n* = 3, *P* = 0.56, GRADE = low].

#### Lateral elbow tendinopathy

**Fig. 2:**
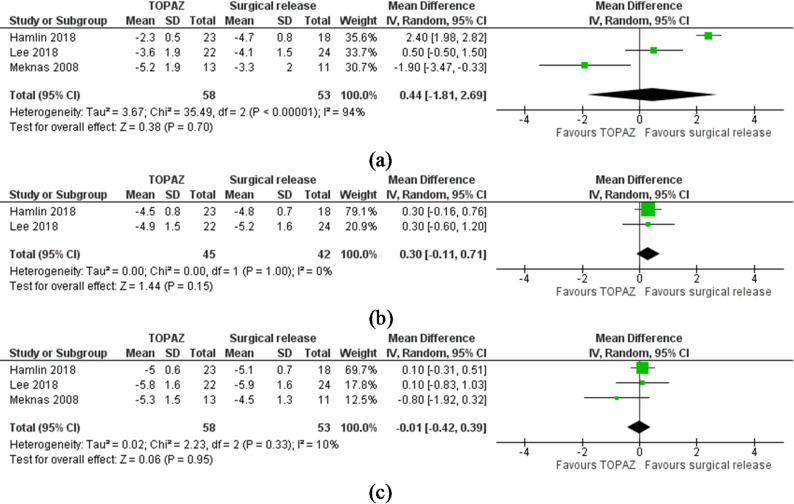
**a** Meta-analysis results showing forest plot and accompanying heterogeneity test for the comparison between radiofrequency microdebridement and open surgical release for lateral elbow tendinopathy for short-term pain visual analogue scale (0–10). **b** Meta-analysis results showing forest plot and accompanying heterogeneity test for the comparison between radiofrequency microdebridement and open surgical release for lateral elbow tendinopathy for mid-term pain visual analogue scale (0–10). **c** Meta-analysis results showing forest plot and accompanying heterogeneity test for the comparison between radiofrequency microdebridement and open surgical release for lateral elbow tendinopathy for long-term pain visual analogue scale (0–10)

Patient-reported pain (VAS): Three studies were included in these meta-analyses [14–16]. Surgical release was open in two and arthroscopic in one study. There were no significant differences between RM and surgical release at any follow-up time period [short-term, MD 0.44 (-1.81 to 2.69), I^2^ = 94%, *P* = 0.70, *n* = 3, GRADE = low, Fig. 2a; mid-term, MD 0.30 (-0.11 to 0.71), I^2^ = 0%, *P* = 0.15, GRADE=moderate, Fig. 2b or long-term, MD − 0.01 (-0.42 to 0.39), I^2^ = 10%, *n* = 3, *P* = 0.95, GRADE=moderate, Fig. 2c. For short-term pain, all studies contributed to the high statistical heterogeneity therefore analyses could not be re-run.

Function (DASH, MEPS): The same three studies were eligible for these meta-analyses [14–16]. RM was statistically superior for short-term and mid-term function but not for long-term function (DASH *n* = 2, MEPS *n* = 1) compared to surgical release [short-term, SMD 0.63 (0.25 to 1.02), I^2^ = 0%, *n* = 3, *P* = 0.001, GRADE=moderate, suppl. Figure 3a; mid-term (DASH), MD -9.18 (-12.36 to -6.00), I^2^ = 0%, *n* = 3, *P* < 0.001, GRADE=moderate, suppl. Figure 3b]. The mid-term difference was also clinically significant. The long-term effects of two interventions were statistically similar for function [MD -7.04 (-16.12 to 2.04), I^2^ = 84%, *n* = 3, *P* = 0.13, GRADE = low, suppl. Figure 3c], and although this difference was clinically significant, the statistical uncertainty (reflected in the wide CI) precludes concluding a definitive effect.

#### Shoulder tendinopathy

**Fig. 3:**
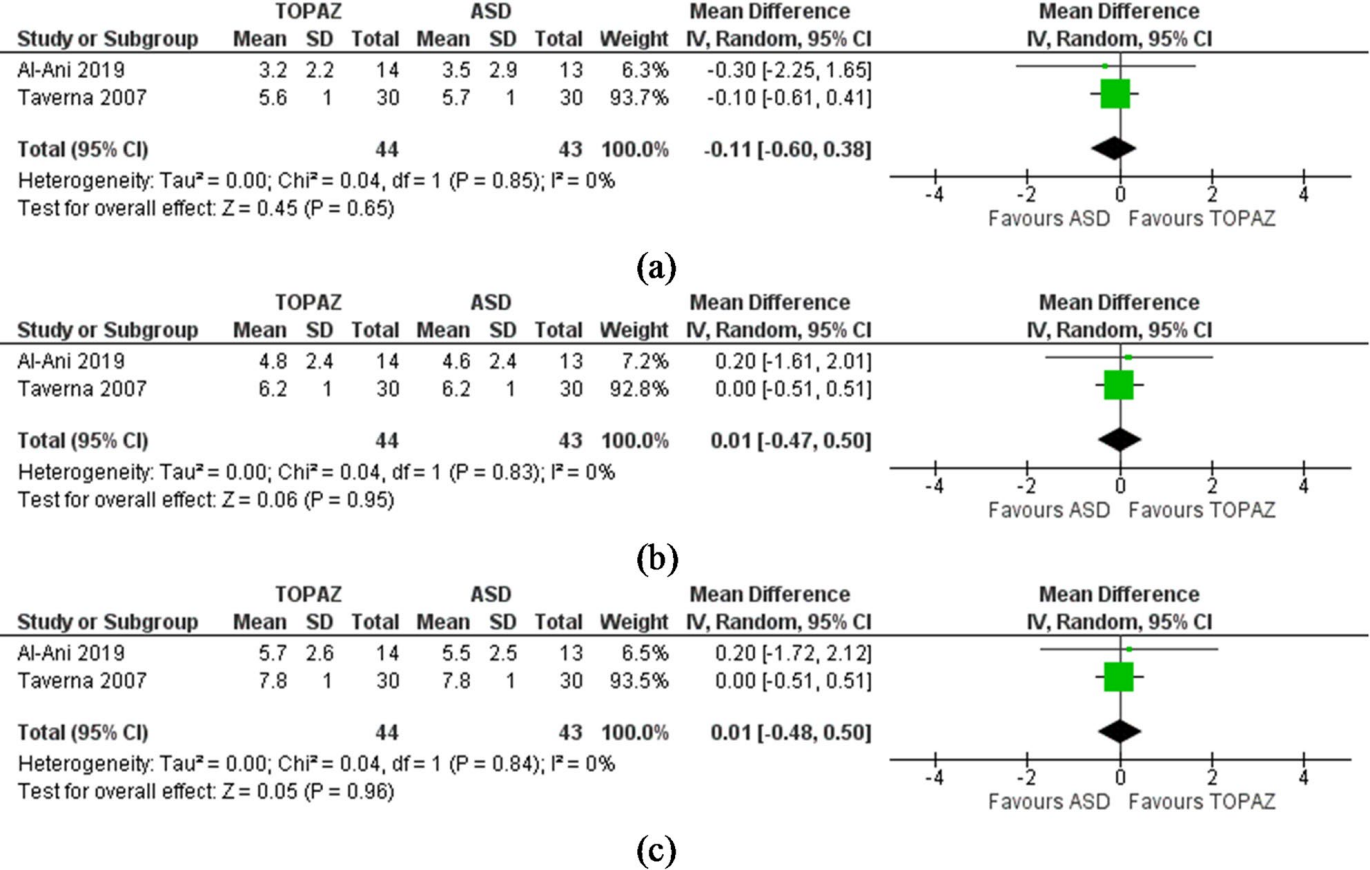
**a** Meta-analysis results showing forest plot and accompanying heterogeneity test for the comparison between radiofrequency microdebridement and arthroscopic subacromial decompression for shoulder tendinopathy for short-term pain visual analogue scale (0–10). **b** Meta-analysis results showing forest plot and accompanying heterogeneity test for the comparison between radiofrequency microdebridement and arthroscopic subacromial decompression for shoulder tendinopathy for mid-term pain visual analogue scale (0–10). **c** Meta-analysis results showing forest plot and accompanying heterogeneity test for the comparison between radiofrequency microdebridement and arthroscopic subacromial decompression for shoulder tendinopathy for long-term pain visual analogue scale (0–10)

Patient-reported pain (VAS): Two studies were included in these meta-analyses [18, 22]. There were no significant differences between RM and arthroscopic subacromial decompression (ASD) at any follow-up time period [short-term, MD − 0.11(-0.60 to 0.38), I^2^ = 0%, *P* = 0.65, GRADE = low, Fig. 3a; mid-term, MD − 0.01, (-0.50 to 0.47), I^2^ = 0%, *P* = 0.95, GRADE = low, Fig. 3b; long-term, MD − 0.01 (-0.50 to 0.5), I^2^ = 0%, *P* = 0.96, GRADE = low, Fig. 3c].

Function (Constant-Murley): The same two RCTs reported functional outcomes. Meta-analyses could not be conducted as only one of the two studies provided variability statistics [22]. This demonstrated no significant differences at any follow-up time period [short-term, MD 3.4 (-9.56 to 16.36), *P* = 0.61; mid-term, MD 7.2 (-3.65 to 18.05), *P* = 0.19; long-term, MD 3.7 (-6.14 to 13.54), *P* = 0.46].

#### Achilles tendinopathy

Patient-reported pain (VAS): A single study, found no statistical significance for mid-term pain between RM or surgical release [MD -0.9(-2.04, 0.24), *P* = 0.12] [20].

Function (VISA-A): The same RCT reported statistically superior increases in VISA-A with RM compared to surgical release at mid-term follow-up [MD 14.30 (2.94 to 25.66), *P* = 0.01, GRADE = low] [20]. This difference was also clinically significant.

### Physiotherapy vs. TOPAZ

#### Achilles tendinopathy

Patient-reported pain (VAS): A single study found statistically and clinically significant long-term pain reduction after RM compared to physiotherapy [MD -3.50 (-4.90 to -2.10), *P* < 0.001, GRADE = low] [19].

Function (FAOS): The same study found no significant differences between the two interventions [MD 9.70 (-4.77 to 24.17), *P* = 0.19] [19].

### Surgery + TOPAZ vs. surgery alone

#### Shoulder tendinopathy

Patient-reported pain (VAS): One study assessed the added effects of RM to arthroscopic subacromial decompression and found no clinically or statistically significant benefits of adding RM to ASD for patient-reported pain at any follow-up time period [short-term, MD 0.10 (-1.04 to 1.24), *P* = 0.86, GRADE = low; mid-term, MD 0.20 (-0.94 to 1.34), *P* = 0.73, GRADE = low; long-term, MD -0.10 (-1.04 to 0.84), *P* = 0.84, GRADE = low] [17].

Function (Constant-Murley): The same study found no short-, mid- or long-term differences in function in the group who had ASD alone and those who had additional RM [MD -3.30 (-12.44 to 5.84), *P* = 0.48; MD 1.90 (-8.63 to 12.43), *P* = 0.72; MD -0.50 (-8.79 to 7.79), *P* = 0.91, respectively, all GRADE = low] [12].

#### Gluteal tendinopathy

Patient-reported pain (VAS): One study found statistically significant long-term benefits in pain when adding RM to gluteal bursectomy versus gluteal bursectomy alone [MD -0.60 (-1.19 to -0.01), *P* = 0.05, GRADE = low]. This difference did not reach clinical significance [21].

Function (mHHS): The same study found no differences in long-term functional improvements with the addition of RM to gluteal bursectomy [MD 0.70 (-9.92 to 11.32), *P* = 0.90, GRADE = low].

### Complications

Eight of the nine RCTs (88.9%) assessed for complications and adverse effects. Four of them [16, 18, 21, 22] observed no complications in either of their treatment groups. Superficial wound infections were reported in a total of three patients in two RCTs [15, 20]; two of them underwent open release for Achilles tendinopathy and one open release for lateral elbow tendinopathy. A study [22] on shoulder tendinopathy that compared arthroscopic subacromial decompression and RM reported adhesive capsulitis in one patient in each group. A patient with lateral tendinopathy treated with RM experienced ongoing swelling and pain secondary to a postoperative haematoma [14]. Finally, a study [20] in Achilles tendinopathy reported the occurrence of a partial tendon rupture in a patient in the RM group which was treated conservatively.

## Discussion

In this systematic review and meta-analysis, we found that RM was associated with largely similar outcomes to surgery for pain relief in Achilles, shoulder and lateral elbow tendinopathies and it may be superior to surgery for function in lateral elbow tendinopathy and Achilles tendinopathy. RM may be superior to physiotherapy for Achilles tendinopathy and it does not appear to confer any added benefits when used to supplement gluteal bursectomy and arthroscopic subacromial decompression. It appears to be safe and well-tolerated. The certainty of evidence was low for most of the results.

Tendinopathy is now recognized as a chronic, degenerative disorder driven by a failed healing response to repetitive mechanical overload rather than a classic inflammatory process. Histopathologic studies demonstrate disruption of the normal parallel collagen architecture, increased type III collagen deposition, mucoid ground substance accumulation, and hypercellularity with rounded tenocytes, features collectively termed angiofibroblastic hyperplasia [23, 24]. These structural changes are accompanied by aberrant neovascularization and ingrowth of sensory nerve fibers, which are believed to contribute to persistent pain and mechanical weakness [24]. At the molecular level, tendinopathic tissue exhibits dysregulated matrix turnover, with elevated expression of matrix metalloproteinases, pro-inflammatory cytokines, and apoptotic mediators that perpetuate extracellular matrix degradation [25]. Emerging evidence further highlights the role of chronic low-grade inflammation and altered mechanotransduction pathways in sustaining tendon degeneration despite the absence of acute inflammatory infiltrates [26]. This maladaptive biological environment creates a cycle in which impaired collagen remodeling and neurovascular proliferation reinforce one another, leading to progressive symptoms and functional decline [27]. Understanding these pathophysiologic mechanisms provides a rationale for biologically targeted interventions such as radiofrequency microablation, which may disrupt neovessels, denervate pathologic tissue, and stimulate a more organized reparative response within the diseased tendon.

After some success using transmyocardial revascularisation and acupuncture in treating ischaemic heart failure, Tasto et al. looked to observe the benefits of RM in treating tendinopathy; specifically, its ability to stimulate angiogenic healing in tendon tissue [28]. Principally, radiofrequency energy ablates soft tissue to promote healing by disruption of covalent molecular bonds [29], resulting in new blood vessel formation. The device itself is applied directly on to the surface of the tendon at a recommended 0.5 s intervals followed by irrigation of sterile saline. Due to its minimally invasive approach and simple application protocol, the TOPAZ wand aims to achieve fewer complications and a quick post operative recovery [30].

Several prospective and retrospective case series have shown the positive effects of RM in Achilles and lateral elbow tendinopathy [28, 31–36]. A comparative cohort study comparing RM with extracorporeal shockwave therapy and eccentric exercise in insertional Achilles tendinopathy showed superior outcomes with RM compared to the other two interventions at long-term follow-up. These results may not be generalisable to non-insertional tendinopathy, however, as the underlying pathophysiology of the two conditions is different [33]. Furthermore, other than tendinopathy, RM has demonstrated promising results in the treatment of plantar fasciitis, however the evidence is even more sparse than in tendinopathy [37].

The limitations of our study are predominantly related to the inherent limitations of the included evidence. Most studies are small and the populations across them are heterogeneous, with the resulting certainty of evidence low in most analyses. Although we have conducted meta-analyses of all tendinopathies combined, those results should be interpreted with caution as different tendinopathies may have different underlying pathophysiology and hence respond to specific interventions differently. For this reason, we did conduct quantitative analyses for each tendinopathy separately. Additionally, none of the included RCTs included a control group with no treatment or sham treatment, therefore the possibility of the improvement seen with the included interventions, including RM, being due to time alone cannot be ruled out. Besides, sham surgery has been shown to be as effective as actual surgery for some tendinopathies in the past [38].

Based on our results and having demonstrated similar outcomes with RM and surgery in the included tendinopathies, we recommend that the use of RM in office settings should be explored as it may be the only way that it is more cost-effective than surgery. Realistically, this would be feasible for lateral elbow and Achilles tendinopathy where local anaesthesia can be utilised thanks to the superficial location of the tendons. Given the implicated costs and the lack of clinical superiority, its use is currently difficult to justify compared to open surgical release of lateral elbow and gluteal tendinopathies; for shoulder tendinopathy, arthroscopic release has important benefits compared to RM, including the minimally invasive approach, the ability to excise the inflamed bursa and perform acromioplasty as well as a diagnostic intra-articular arthroscopy, therefore we do not recommend the use of RM for shoulder tendinopathy. Formal cost analyses should be assessed in the future for definitive conclusions. Finally, the low to moderate certainty of evidence along with the lack of placebo-or sham-controlled studies should be taken into account when interpreting the results as, without those, the actual benefits conferred by RM as an intervention cannot be proven.

## Conclusion

This study found that RM appears to be associated with similar pain outcomes to surgical release in lateral elbow, shoulder, and Achilles tendinopathies, with possible short- to mid-term functional advantages in lateral elbow and Achilles tendinopathy. Evidence from individual studies also suggests potential benefits over physiotherapy in Achilles tendinopathy, while adding RM to surgical procedures does not appear to improve outcomes. RM was generally safe and well tolerated. However, the current evidence base is generally of low quality, limited by small sample sizes, high risk of bias, and the absence of sham-controlled trials. Larger, high-quality randomised studies with longer follow-up are required to clarify the clinical role and cost-effectiveness of RM in the management of tendinopathy.

## Supplementary Information

Below is the link to the electronic supplementary material.

Supplementary Material 1

## Data Availability

all data are available upon request from the senior author.

## References

[1] Millar NL, Silbernagel KG, Thorborg K et al. Tendinopathy Nat Reviews Disease Primers. 2021. 7(1). 10.1038/s41572-020-00234-1 33414454

[2] Riley G. Tendinopathy—from basic science to treatment. Nat Clin Pract Rheumatol. 2008;4(2):82–9. 18235537 10.1038/ncprheum0700

[3] Abate M, Silbernagel K, Siljeholm C, et al. Pathogenesis of tendinopathies: inflammation or degeneration? Arthritis Res Therapy. 2009;11(3):235. 10.1186/ar2723 PMC2714139 19591655

[4] Maffulli N. New options in the management of tendinopathy. Open Access J Sports Med. 2010; 29. 10.2147/oajsm.s7751 PMC3781852 24198540

[5] Andres BM, Murrell GAC. Treatment of Tendinopathy: What Works, What Does Not, and What Is on the Horizon. Clin Orthop Relat Res. 2008;466(7):1539–54. 18446422 10.1007/s11999-008-0260-1 PMC2505250

[6] Page MJ, McKenzie JE, Bossuyt PM, et al. The PRISMA 2020 statement: an updated guideline for reporting systematic reviews. BMJ. 2021;372:n71. 33782057 10.1136/bmj.n71 PMC8005924

[7] Higgins JP, Altman DG, Gøtzsche PC, Cochrane Bias Methods Group; Cochrane Statistical Methods Group, et al. The Cochrane Collaboration’s tool for assessing risk of bias in randomised trials. BMJ. 2011;343:d5928. 22008217 10.1136/bmj.d5928 PMC3196245

[8] Guyatt GH, Oxman AD, Vist GE, Kunz R, Falck-Ytter Y, Alonso-Coello P, Schünemann HJ, GRADE Working Group. GRADE: an emerging consensus on rating quality of evidence and strength of recommendations. BMJ. 2008;336(7650):924–6. 18436948 10.1136/bmj.39489.470347.AD PMC2335261

[9] Schünemann H, Brożek J, Guyatt G, Oxman A, editors. *GRADE Handbook for Grading Quality of Evidence and Strength of Recommendations*. Updated October 2013. The GRADE Working Group, 2013. Available at guidelinedevelopment.org/handbook.

[10] Clement N, Bardgett M, Weir D, Holland J, Gerrand J, Deehan D. What is the Minimum Clinically Important Difference for the WOMAC Index After TKA? Clin Orthop Relat Res. 2018;476(10):2005–14. 30179956 10.1097/CORR.0000000000000444 PMC6259858

[11] Desai S, Peterson AJ, Wing K, et al. Minimally Important Difference in the Foot and Ankle Outcome Score Among Patients Undergoing Hallux Valgus Surgery. Foot Ankle Int. 2019;40(6):694–701. 30873859 10.1177/1071100719831392

[12] Cohen J. Statistical Power Analysis for the Behavioral Sciences. 2nd ed. Hillsdale, NJ: Lawrence Erlbaum Associates,; 1988.

[13] Meknas K, Al Hassoni TN, Odden-Miland Å, Castillejo M, Kartus J. Medium-Term Results After Treatment of Recalcitrant Lateral Epicondylitis. Orthop J Sports Med. 2013;1(4):232596711350543. 10.1177/2325967113505433 PMC4555492 26535247

[14] Lee JH, Park I, Hyun HS, Shin SJ. A Comparison of Radiofrequency-Based Microtenotomy and Arthroscopic Release of the Extensor Carpi Radialis Brevis Tendon in Recalcitrant Lateral Epicondylitis: A Prospective Randomized Controlled Study. Arthroscopy: J Arthroscopic Relat Surg. 2018;34(5):1439–46. 10.1016/j.arthro.2017.11.029 29366739

[15] Hamlin K, Munro C, Barker SL, McKenna S, Kumar K. Open release versus radiofrequency microtenotomy in the treatment of lateral epicondylitis: a prospective randomized controlled trial. Shoulder Elb. 2017;10(1):45–51. 10.1177/1758573217715255 PMC5734528 29276537

[16] Meknas K, Odden-Miland Å, Mercer JB, Castillejo M, Johansen O. Radiofrequency microtenotomy: a promising method for treatment of recalcitrant lateral epicondylitis. Am J Sports Med. 2008;36(10):1960–5. 18559469 10.1177/0363546508318045

[17] Lu Y, Zhang Q, Zhu Y, Jiang C. Is radiofrequency treatment effective for shoulder impingement syndrome? A prospective randomized controlled study. J Shoulder Elbow Surg. 2013;22(11):1488–94. 23994459 10.1016/j.jse.2013.06.006

[18] Taverna E, Battistella F, Sansone V, Perfetti C, Tasto JP. Radiofrequency-Based Plasma Microtenotomy Compared With Arthroscopic Subacromial Decompression Yields Equivalent Outcomes for Rotator Cuff Tendinosis. Arthrosc J Arthroscopic Relat injury. 2007;23(10):1042–51. 10.1016/j.arthro.2007.04.018 17916468

[19] Al-Ani Z, Meknas D, Kartus JT, Lyngedal Ø, Meknas K. Radiofrequency Microtenotomy or Physical Therapy for Achilles Tendinopathy: Results of a Randomized Clinical Trial. Orthop J Sports Med. 2021;9(12):232596712110625. 10.1177/23259671211062555 PMC8721382 34988234

[20] Morrison RJM, Brock TM, Reed MR, Muller SD. Radiofrequency Microdebridement Versus Surgical Decompression for Achilles Tendinosis: A Randomized Controlled Trial. The Journal of Foot and Ankle Surgery: Official Publication of the American College of Foot and Ankle Surgeons. 2017; 56(4):708–12. 10.1053/j.jfas.2017.01.049 28495412

[21] Blakey CM, O’Donnell J, Klaber I, et al. Radiofrequency Microdebridement as an Adjunct to Arthroscopic Surgical Treatment for Recalcitrant Gluteal Tendinopathy: A Double-Blind, Randomized Controlled Trial. Orthop J Sports Med. 2020;8(1):232596711989560. 10.1177/2325967119895602 PMC6984435 32047828

[22] Al-Ani Z, Wergeland JE, Kartus J-T, Knutsen G, Meknas K. Radiofrequency microtenotomy: a promising method for treatment of rotator cuff tendinopathy. Knee surg Sports Traumatol Arthrosc. 2019;27(12):3856–63. 31473769 10.1007/s00167-019-05689-8

[23] Maffulli N, Khan KM, Puddu G. Overuse tendon conditions: time to change a confusing terminology. Arthroscopy. 1998;14(8):840–3. 9848596 10.1016/s0749-8063(98)70021-0

[24] Sharma P, Maffulli N. Tendon injury and tendinopathy: healing and repair. J Bone Joint Surg Am. 2005;87(1):187–202. 15634833 10.2106/JBJS.D.01850

[25] Chisari E, Rehak L, Khan WS, Maffulli N. Tendon healing in presence of chronic low-level inflammation: a systematic review. Br Med Bull. 2019;132(1):97–116. 31838495 10.1093/bmb/ldz035

[26] Citeroni MR, Ciardulli MC, Russo V, Della Porta G, Mauro A, El Khatib M, Di Mattia M, Galesso D, Barbera C, Forsyth NR, Maffulli N, Barboni B. In Vitro Innovation of Tendon Tissue Engineering Strategies. Int J Mol Sci. 2020;21(18):6726. 32937830 10.3390/ijms21186726 PMC7555358

[27] Maffulli N, Cuozzo F, Migliorini F, Oliva F. The tendon unit: biochemical, biomechanical, hormonal influences. J Orthop Surg Res. 2023;18(1):311. 37085854 10.1186/s13018-023-03796-4 PMC10120196

[28] Tasto JP, Cummings JL, Medlock V, Hardesty R, Amiel D. Microtenotomy Using a Radiofrequency Probe to Treat Lateral Epicondylitis.Arthroscopy. J Arthroscopic Relat injury. 2005;21(7):851–60. 10.1016/j.arthro.2005.03.019 16012499

[29] Png W, Koo K. Open vs Percutaneous TOPAZ Coblation for the Management of Plantar Fasciitis: Comparison of the Two Techniques in Obese Patients. J Foot Ankle Surg (Asia Pacific). 2020;7(1):14–20.

[30] ArthroCare Corporation. 510 (k) Summary ArthroCare Coperation, Topaz ArthroWand General Information [Internet] c2008,[updated Feb2008, cited 2024 May 15]. Available from: https://www.accessdata.fda.gov/cdrh_docs/pdf5/K053567.pdf

[31] Sarimo J, Orava S. Fascial incision and adhesiolysis combined with radiofrequency microtenotomy in treatment of chronic midportion Achilles tendinopathy. Scand J Surg. 2011;100(2):125–8. 21737390 10.1177/145749691110000211

[32] Yeap EJ, Chong KW, Singh Rikhraj I. Radiofrequency coblation for chronic foot and ankle tendinosis. J Orthop Surg (Hong Kong). 2009;17(3):325–30. 20065374 10.1177/230949900901700317

[33] Wei M, Liu Y, Li Z, Wang Z. Comparison of Clinical Efficacy Among Endoscopy-Assisted Radio-Frequency Ablation, Extracorporeal Shockwaves, and Eccentric Exercises in Treatment of Insertional Achilles Tendinosis. J Am Podiatr Med Assoc. 2017;107(1):11–6. 27723374 10.7547/14-146

[34] Shibuya N, Thorud JC, Humphers JM, Devall JM, Jupiter DC. Is percutaneous radiofrequency coblation for treatment of Achilles tendinosis safe and effective? J Foot Ankle Surg. 2012;51(6):767–71. 22974813 10.1053/j.jfas.2012.08.011

[35] Tasto JP, Richmond JM, Cummings JR, Hardesty R, Amiel D. Radiofrequency Microtenotomy for Elbow Epicondylitis: Midterm Results. Am J Orthop (Belle Mead NJ). 2016;45(1):29–33. 26761915

[36] Viswanathan S, Kashyap Shanker H. Long-Term Functional Outcomes Following Radiofrequency Microtenotomy for Lateral Epicondylitis of Elbow. Cureus. 2022;14(10):e30317. 36407210 10.7759/cureus.30317 PMC9661452

[37] Thor J, Mao DW, Chandrakumara D, Zheng Q, Wook Yoo T, King CKK. Radiofrequency microtenotomy for plantar fasciitis: A systematic review and meta-analysis. Foot (Edinb). 2022;50:101869. 35219133 10.1016/j.foot.2021.101869

[38] Challoumas D, Clifford C, Kirwan P, Millar NL. How does surgery compare to sham surgery or physiotherapy as a treatment for tendinopathy? A systematic review of randomised trials. BMJ Open Sport Exerc Med. 2019;5(1):e000528. 31191975 10.1136/bmjsem-2019-000528 PMC6539146

