# Supplementary material for: Efficacy of radiofrequency microdebridement (TOPAZ) in tendinopathy: a systematic review and meta-analysis of randomised clinical trials
Source: J Orthop Surg Res. 2026 Mar 31;21:302. doi: 10.1186/s13018-026-06827-y (PMC13162353; doi:10.1186/s13018-026-06827-y)
Supplement: Supplementary file 1 — Supplementary Material 1 [file 13018_2026_6827_MOESM1_ESM.docx]

**Supplementary material**

**Figures**


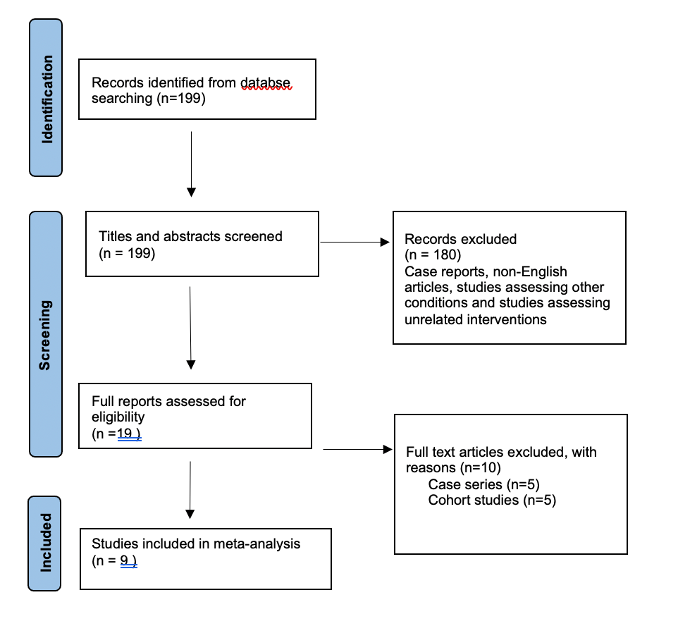


**Suppl. Figure 1.** PRISMA flow diagram summarising the article selection process.


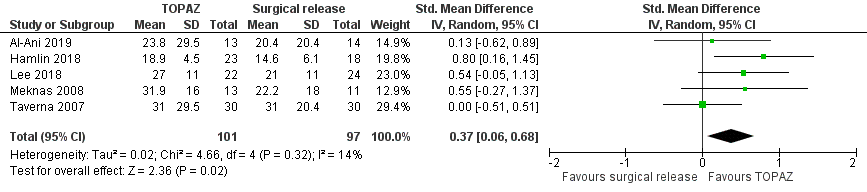


**Suppl. Figure 2a.** Meta-analysis results showing forest plot and accompanying heterogeneity test for the comparison between radiofrequency microdebridement and surgical release (open or arthroscopic) for all tendinopathies combined for short-term function (all functional instruments combined).


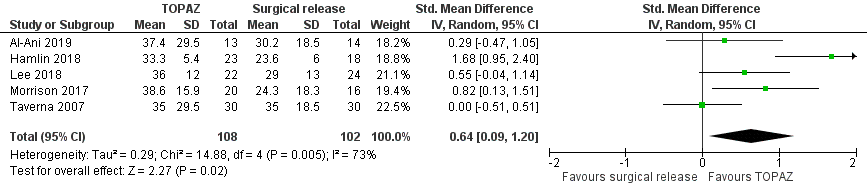


**Suppl. Figure 2b.** Meta-analysis results showing forest plot and accompanying heterogeneity test for the comparison between radiofrequency microdebridement and surgical release (open or arthroscopic) for all tendinopathies combined for mid-term function (all functional instruments combined).


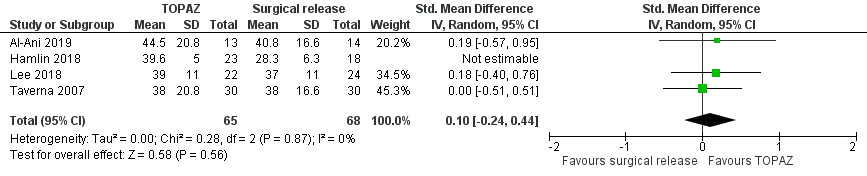


**Suppl. Figure 2c.** Meta-analysis results showing forest plot and accompanying heterogeneity test for the comparison between radiofrequency microdebridement and surgical release (open or arthroscopic) for all tendinopathies combined for long-term function (all functional instruments combined).


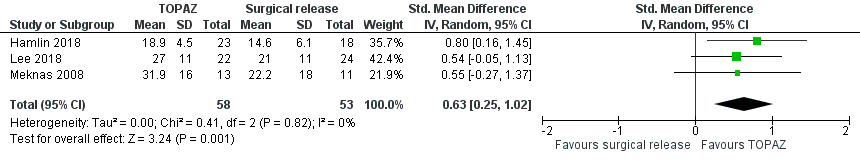


**Suppl. Figure 3a.** Meta-analysis results showing forest plot and accompanying heterogeneity test for the comparison between radiofrequency microdebridement and open surgical release for lateral elbow tendinopathy for short-term function DASH, 0-100).


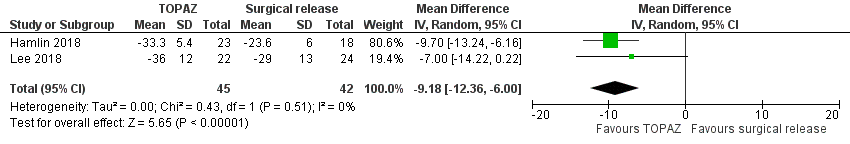


**Suppl. Figure 3b.** Meta-analysis results showing forest plot and accompanying heterogeneity test for the comparison between radiofrequency microdebridement and open surgical release for lateral elbow tendinopathy for mid-term function DASH, 0-100).


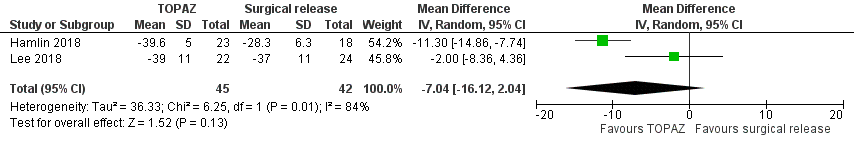


**Suppl. Figure 3c.** Meta-analysis results showing forest plot and accompanying heterogeneity test for the comparison between radiofrequency microdebridement and open surgical release for lateral elbow tendinopathy for long-term function DASH, 0-100).

**Tables**

|  | Selection Bias | | Performance Bias | Detection Bias | Attrition Bias | Reporting Bias | **Overall Risk of Bias** |
| --- | --- | --- | --- | --- | --- | --- | --- |
| Paper | Random Sequence Generation | Allocation Concealment | Blinding of participants and personnel | Blinding of outcome assessment | Incomplete outcome data | Selective Reporting |  |
| Al-Ani 2021 | Low | Low | High | High | Low | Low | **High** |
| Blakey 2020 | Low | Unclear | High | High | Low | Low | **High** |
| Hamlin 2018 | Low | High | High | High | High | Low | **High** |
| Lee 2018 | Low | Low | High | High | High | Low | **High** |
| Lu 2013 | Low | High | High | High | High | Low | **High** |
| Meknas 2008 | Unclear | Unclear | High | High | Unclear | Low | **High** |
| Meknas 2013 | Unclear | Unclear | High | High | High | Low | **High** |
| Morrison 2017 | Unclear | High | High | High | Low | Low | **High** |
| Taverna 2007 | Unclear | Unclear | High | High | Unclear | High | **High** |

**Suppl. Table 1.** Risk of bias assessment of the included studies using the Cochrane Collaboration tool (version 1)

| **Section and Topic** | **Item #** | **Checklist item** | **Location where item is reported** |
| --- | --- | --- | --- |
| **TITLE** | | |  |
| Title | 1 | Identify the report as a systematic review. | Title |
| **ABSTRACT** | | |  |
| Abstract | 2 | See the PRISMA 2020 for Abstracts checklist. | Abstract |
| **INTRODUCTION** | | |  |
| Rationale | 3 | Describe the rationale for the review in the context of existing knowledge. | Lines 63-75 |
| Objectives | 4 | Provide an explicit statement of the objective(s) or question(s) the review addresses. | Lines 71-76 |
| **METHODS** | | |  |
| Eligibility criteria | 5 | Specify the inclusion and exclusion criteria for the review and how studies were grouped for the syntheses. | Lines 84-88 |
| Information sources | 6 | Specify all databases, registers, websites, organisations, reference lists and other sources searched or consulted to identify studies. Specify the date when each source was last searched or consulted. | Lines 91-102 |
| Search strategy | 7 | Present the full search strategies for all databases, registers and websites, including any filters and limits used. | Lines 91-102 |
| Selection process | 8 | Specify the methods used to decide whether a study met the inclusion criteria of the review, including how many reviewers screened each record and each report retrieved, whether they worked independently, and if applicable, details of automation tools used in the process. | Lines 105-109 |
| Data collection process | 9 | Specify the methods used to collect data from reports, including how many reviewers collected data from each report, whether they worked independently, any processes for obtaining or confirming data from study investigators, and if applicable, details of automation tools used in the process. | Lines 112-114 |
| Data items | 10a | List and define all outcomes for which data were sought. Specify whether all results that were compatible with each outcome domain in each study were sought (e.g. for all measures, time points, analyses), and if not, the methods used to decide which results to collect. | Lines 115-118 |
|  | 10b | List and define all other variables for which data were sought (e.g. participant and intervention characteristics, funding sources). Describe any assumptions made about any missing or unclear information. | Table 1 |
| Study risk of bias assessment | 11 | Specify the methods used to assess risk of bias in the included studies, including details of the tool(s) used, how many reviewers assessed each study and whether they worked independently, and if applicable, details of automation tools used in the process. | Lines 124-125 |
| Effect measures | 12 | Specify for each outcome the effect measure(s) (e.g. risk ratio, mean difference) used in the synthesis or presentation of results. | Lines 133-136 |
| Synthesis methods | 13a | Describe the processes used to decide which studies were eligible for each synthesis (e.g. tabulating the study intervention characteristics and comparing against the planned groups for each synthesis (item #5)). | Lines 112-114 |
|  | 13b | Describe any methods required to prepare the data for presentation or synthesis, such as handling of missing summary statistics, or data conversions. | N/A |
|  | 13c | Describe any methods used to tabulate or visually display results of individual studies and syntheses. | Lines 131-136 |
|  | 13d | Describe any methods used to synthesize results and provide a rationale for the choice(s). If meta-analysis was performed, describe the model(s), method(s) to identify the presence and extent of statistical heterogeneity, and software package(s) used. | Lines 131-136 |
|  | 13e | Describe any methods used to explore possible causes of heterogeneity among study results (e.g. subgroup analysis, meta-regression). | N/A |
|  | 13f | Describe any sensitivity analyses conducted to assess robustness of the synthesized results. | Lines 134-136 |
| Reporting bias assessment | 14 | Describe any methods used to assess risk of bias due to missing results in a synthesis (arising from reporting biases). | N/A |
| Certainty assessment | 15 | Describe any methods used to assess certainty (or confidence) in the body of evidence for an outcome. | Lines 125-128 |
| **RESULTS** | | |  |
| Study selection | 16a | Describe the results of the search and selection process, from the number of records identified in the search to the number of studies included in the review, ideally using a flow diagram. | Lines 138-146 |
|  | 16b | Cite studies that might appear to meet the inclusion criteria, but which were excluded, and explain why they were excluded. | N/A |
| Study characteristics | 17 | Cite each included study and present its characteristics. | Table 1 |
| Risk of bias in studies | 18 | Present assessments of risk of bias for each included study. | Suppl table 1 |
| Results of individual studies | 19 | For all outcomes, present, for each study: (a) summary statistics for each group (where appropriate) and (b) an effect estimate and its precision (e.g. confidence/credible interval), ideally using structured tables or plots. | Figures 1-3, suppl figures 2-4 |
| Results of syntheses | 20a | For each synthesis, briefly summarise the characteristics and risk of bias among contributing studies. | Table 1, suppl table 1 |
|  | 20b | Present results of all statistical syntheses conducted. If meta-analysis was done, present for each the summary estimate and its precision (e.g. confidence/credible interval) and measures of statistical heterogeneity. If comparing groups, describe the direction of the effect. | Figures 1-3, suppl figures 2-4 |
|  | 20c | Present results of all investigations of possible causes of heterogeneity among study results. | Lines 164-166, lines 177-180 |
|  | 20d | Present results of all sensitivity analyses conducted to assess the robustness of the synthesized results. | Lines 164-166, lines 177-180 |
| Reporting biases | 21 | Present assessments of risk of bias due to missing results (arising from reporting biases) for each synthesis assessed. | N/A |
| Certainty of evidence | 22 | Present assessments of certainty (or confidence) in the body of evidence for each outcome assessed. | Throughout Results section |
| **DISCUSSION** | | |  |
| Discussion | 23a | Provide a general interpretation of the results in the context of other evidence. | Lines 266-272 |
|  | 23b | Discuss any limitations of the evidence included in the review. | Lines 291-304 |
|  | 23c | Discuss any limitations of the review processes used. | Lines 291-304 |
|  | 23d | Discuss implications of the results for practice, policy, and future research. | Lines 305-323 |
| **OTHER INFORMATION** | | |  |
| Registration and protocol | 24a | Provide registration information for the review, including register name and registration number, or state that the review was not registered. | Lines 80-81 |
|  | 24b | Indicate where the review protocol can be accessed, or state that a protocol was not prepared. | Lines 80-81 |
|  | 24c | Describe and explain any amendments to information provided at registration or in the protocol. | N/A |
| Support | 25 | Describe sources of financial or non-financial support for the review, and the role of the funders or sponsors in the review. | N/A |
| Competing interests | 26 | Declare any competing interests of review authors. | Title page |
| Availability of data, code and other materials | 27 | Report which of the following are publicly available and where they can be found: template data collection forms; data extracted from included studies; data used for all analyses; analytic code; any other materials used in the review. | Title page |

*From:*  Page MJ, McKenzie JE, Bossuyt PM, Boutron I, Hoffmann TC, Mulrow CD, et al. The PRISMA 2020 statement: an updated guideline for reporting systematic reviews. BMJ 2021;372:n71. doi: 10.1136/bmj.n71. This work is licensed under CC BY 4.0. To view a copy of this license, visit <https://creativecommons.org/licenses/by/4.0/>
